# Ellagic Acid and Urolithins A and B Differentially Regulate Fat Accumulation and Inflammation in 3T3-L1 Adipocytes While Not Affecting Adipogenesis and Insulin Sensitivity

**DOI:** 10.3390/ijms21062086

**Published:** 2020-03-18

**Authors:** Luis Cisneros-Zevallos, Woo Young Bang, Claudia Delgadillo-Puga

**Affiliations:** 1 Department of Horticultural Sciences, Texas A&M University, College Station, TX 77843-2133, USA; wybang@gmail.com; 2 Department of Nutrition and Food Science, Texas A&M University, College Station, TX 77843, USA; 3 Departamento de Nutrición Animal Dr. Fernando Pérez-Gil Romo, Instituto Nacional de Ciencias Médicas y Nutrición Salvador Zubirán (INCMNSZ), CDMX 14080, Mexico; claudia.delgadillop@incmnsz.mx

**Keywords:** urolithins A and B, ellagic acid, adipogenesis, lipid metabolism, inflammation, insulin sensitivity, 3T3-L1 adipocytes

## Abstract

Ellagic acid (EA) is a component of ellagitannins, present in crops such as pecans, walnuts, and many berries, which metabolized by the gut microbiota forms urolithins A, B, C, or D. In this study, ellagic acid, as well as urolithins A and B, were tested on 3T3-L1 preadipocytes for differentiation and lipid accumulation. In addition, inflammation was studied in mature adipocytes challenged with lipopolysaccharide (LPS). Results indicated that EA and urolithins A and B did not affect differentiation (adipogenesis) and only EA and urolithin A attenuated lipid accumulation (lipogenesis), which seemed to be through gene regulation of glucose transporter type 4 (GLUT4) and adiponectin. On the other hand, gene expression of cytokines and proteins associated with the inflammation process indicate that urolithins and EA differentially inhibit tumor necrosis factor alpha (TNFα), inducible nitric oxide synthase (iNOS), interleukin 6 (IL-6), and monocyte chemoattractant protein-1 (MCP-1). Urolithins A and B were found to reduce nuclear levels of phosphorylated nuclear factor κB (p-NF-κB), whereas all treatments showed expression of nuclear phosphorylated protein kinase B (p-AKT) in challenged LPS cells when treated with insulin, indicating the fact that adipocytes remained insulin sensitive. In general, urolithin A is a compound able to reduce lipid accumulation, without affecting the protein expression of peroxisome proliferator-activated receptor-γ (PPARγ), CCAAT/enhancer binding protein-α (c/EBPα), and PPARα, whereas EA and urolithin B were found to enhance PPARγ and c/EBPα protein expressions as well as fatty acid (FA) oxidation, and differentially affected lipid accumulation.

## 1. Introduction

Ellagic acid (EA) is present in plants in the form of hydrozable tannins called ellagitannins with protective roles in the plant cell and possibly regulating plant metabolism. Different sources of ellagic acid include pecans, walnuts, and many berries [1,2,3]. Ellagic acid is extensively metabolized by the gut microbiota to yield the hydroxy-dibenzopyranone derivatives, used to form urolithins A, B, C, or D. Urolithins (Uro) are bioavailable, and they can reach a concentration as high as a 0.5–18.5 μM level as glucuronide derivatives in plasma after consuming 1L of pomegranate juice daily [4,5], or up to 185 μM after consuming 200g of walnuts [6]. However, there are urolithin non-producer individuals (metabotype 0), as well as urolithin producer individuals metabotypes A (mainly urolithin A derivatives) or metabotype B (mainly urolithin B and A derivatives). Differences in intestinal microbiota have been associated with the interindividual variability, which seems also correlated with health status and predisposition to chronic diseases (e.g., metabolic syndrome) [7].

Several studies have indicated that ellagic acid and its derivative urolithins possess beneficial effects against prostate and colon cancer and exert some proestrogenic/antiestrogenic effects [8,9,10], as well as having anti-inflammatory activity in colon fibroblasts and protection against oxidative stress [11,12]. Other reports have shown antiatherogenic effects in human umbilical vein endothelial cells [13] and neuroprotective effects against Alzheimer’s disease in in vitro studies [14].

However, these compounds (EA and Uro) as potential anti-obesity treatments have started to acquire attention in recent years. Obesity is a complex disorder with multiple causes, including both genetic and environmental factors, and is associated with the risk of insulin resistance (type 2 diabetes), fatty liver disease, hypertension, and atherosclerosis [15].

Over the past years, in vitro systems have been used to define the transcriptional events regulating preadipocyte differentiation (adipogenesis) and adipocyte function; 3T3-L1 being the most frequently employed cell line [16]. During the differentiation process of preadipocytes into adipocytes, an elaborate network of transcription factors coordinates the expression of hundreds of specific proteins. At the center of this elaborate network are specific adipogenic factors such as peroxisome proliferator-activated receptor-γ (PPARγ) and the CCAAT/enhancer binding protein-α (c/EBPα). Both transcription factors are key to successfully complete the entire differentiation process. PPARγ is a member of the nuclear–receptor superfamily—its expression induces growth arrest and initiates adipogenesis in fibroblasts. It is also required for maintenance of the differentiated state [17], and in particular is considered the master regulator of adipogenesis; without it, precursor cells are incapable of expressing any known aspect of the adipocyte phenotype [18]. On the other hand, cells deficient in c/EBPα are capable of adipocyte differentiation; however, these c/EBPα-deficient cells are insulin resistant [19,20]. More recently, data from a variety of knockout mice have confirmed these in vitro studies, showing that many components of this network are required regulators of adipocyte development and function. Another transcription factor member of nuclear-receptor superfamily, PPARα, plays an important role in fatty acid oxidation in liver and skeletal muscle, and it is reported that PPARα activators may affect adipose tissue metabolism and the activation of both mitochondrial and peroxisomal β-oxidation [21,22].

**Figure 1:**
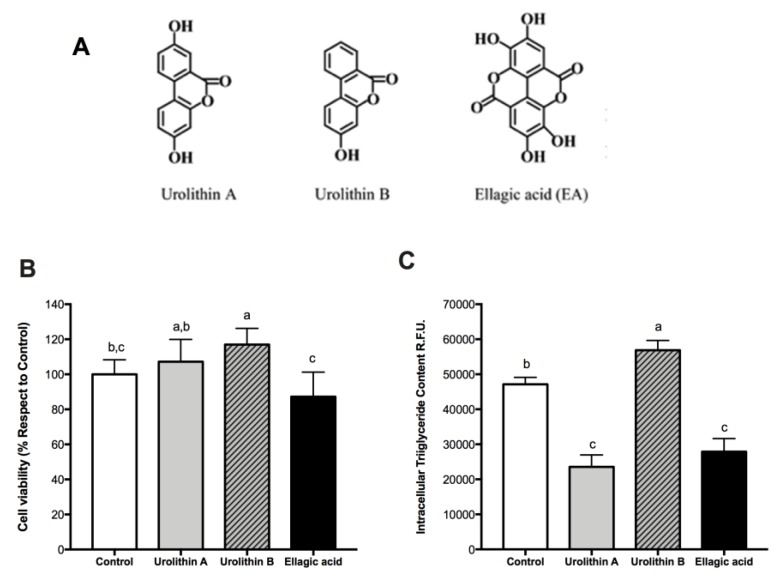
The effect of ellagic acid, urolithin A, and urolithin B on cell viability and intracellular triglyceride content in preadipocytes and mature adipocytes. (**A**) Chemical structures of ellagic acid and its derivatives urolithins A and B. (**B**) Cell viability of 3T3-L1 preadipocytes in control samples (0.025% dimethyl sulfoxide (DMSO)) and after 24 h treatment with 25 μM urolithin A, urolithin B, and ellagic acid. The values represent the mean ± SD (*n* = 3) of three independent experiments conducted in duplicate. (**C**) Intracellular lipid accumulation in 3T3-L1 mature adipocytes after 8-day treatment with 25 μM of urolithin A, urolithin B, ellagic acid, and control (0.025% DMSO). Data are means ± SD (*n* = 3) of three independent experiments conducted in triplicate. Different letters among bars denote significant changes among treatments and control (*p* ≤ 0.05) performed by ANOVA and Tukey’s post hoc test.

The objective of this study was to determine the effect of ellagic acid and derived gut microbial metabolites urolithins A and B (Figure 1A) on adipocyte differentiation in murine 3T3-L1 fibroblasts and fat accumulation. The study also examined the effect on PPARγ, PPARα, and c/EBPα, and genes associated with lipogenic and lipolytic enzymes in order to provide basic data for the elucidation of the mode of action. In addition, we reported the anti-inflammatory effects of these compounds on lipopolysaccharide (LPS)-challenged adipocytes and insulin-sensitizing activities. This work is based on a report by Cisneros-Zevallos and Bang [23], which, to the best of our knowledge, was the first study on ellagic acid-derived gut microbial metabolites urolithins A and B’s effects on adipocytes.

## 2. Results and Discussion

### 2.1. Effect of Ellagic Acid and Urolithins A and B on Cell Viability

To study the effect on cell viability, 3T3-L1 preadipocytes were treated with ellagic acid and urolithins at 25 μM for 24 h; then the MTS assay was performed. No significant reductions were observed with respect to the control after the treatments with ellagic acid and urolithins A and B (Figure 1B). These results showed that these compounds did not affect cell viability in preadipocytes. Similar results were reported in human colonic fibroblasts by González-Sarrías et al. [12].

### 2.2. Effect of Ellagic Acid and Urolithins A and B on Intracellular Triglyceride Content

To investigate effects of ellagic acid and derived metabolites, urolithins A and B, on intracellular triglyceride accumulation during the differentiation of preadipocytes into fully mature adipocytes (day 8), cells were cultured with these compounds at a concentration of 25 μM from day 0 to day 8. AdipoRed assay was performed to measure the amount of intracellular triglycerides. We found a significant decrease over intracellular triglyceride accumulation with ellagic acid and urolithin A (*p* < 0.05), whereas with urolithin B, a significant increase was observed (Figure 1C). These results showed a differential effect of both urolithins, likely due to differences between their chemical structures (Figure 1A). Moreover, this result could have been due to anti-adipogenic, anti-lipogenic, or enhanced lipolytic effects of ellagic acid and urolithin A. To answer this question, PPARγ and c/EBPα protein expression was measured.

### 2.3. Effect of Ellagic Acid and Urolithins A and B on Transcription Factors Related to Adipocyte Differentiation

**Figure 2:**
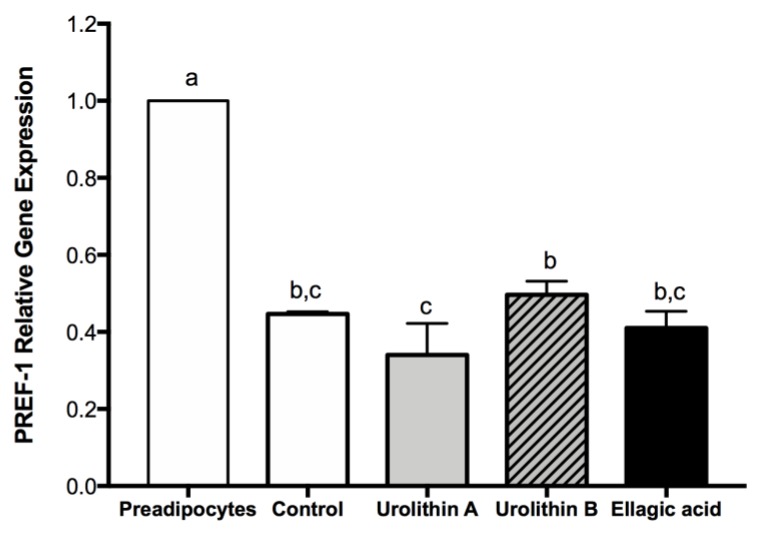
Effect of ellagic acid, urolithin A, and urolithin B over preadipocyte factor 1 (PREF-1) gene expression at day 4 after differentiation induction. 3T3-L1 cells were treated with 25 μM of each compound. For control samples, 0.025% DMSO was used. The values represent the mean ± SD (*n* = 3) of three independent experiments. Different letters among bars denote significant changes among treatments and control (*p* ≤ 0.05) performed by ANOVA and Tukey’s post hoc test.

**Figure 3:**
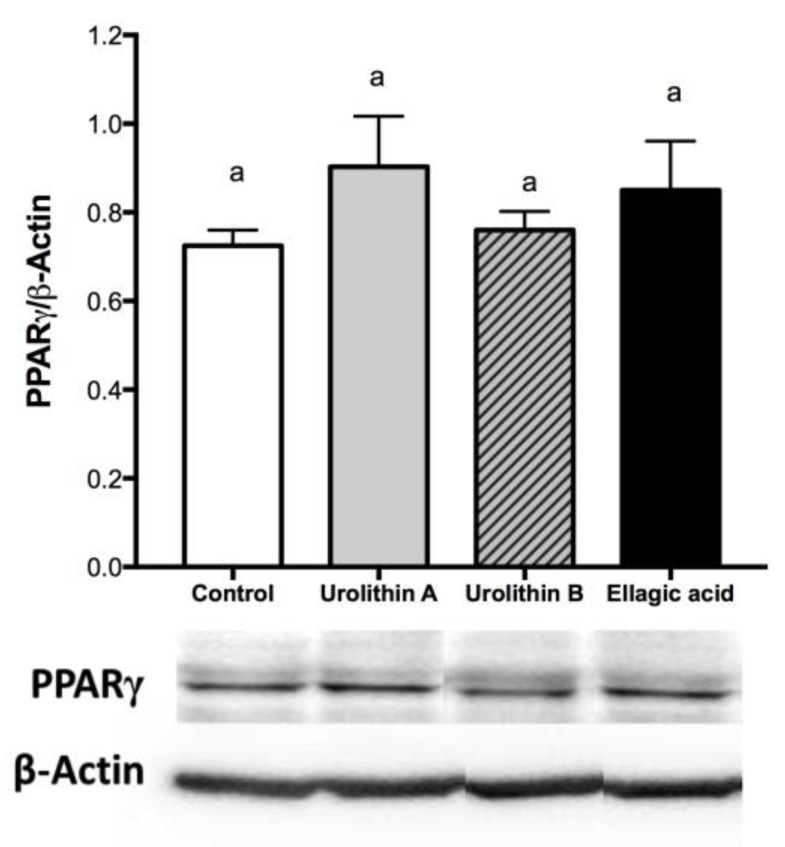
Effect of ellagic acid, urolithin A, and urolithin B over PPARγ protein expression at day 4 after differentiation induction. 3T3-L1 cells were treated with 25 μM of each compound. For control samples, 0.025% DMSO was used. The values represent the mean ± SD (*n* = 3) of three independent experiments. Different letters among bars denote significant changes among treatments and control (*p* ≤ 0.05) performed by ANOVA and Tukey’s post hoc test.

In the early stage of differentiation, preadipocyte factor 1 (PREF-1) is an important transcription factor present only in preadipocytes, and the overexpression of this marker has an inhibitory effect on the differentiation process. On the other hand, PPARγ is a key transcription factor to conduct the differentiation process in preadipocytes, and the expression of this marker starts when differentiation is triggered, being maintained during the whole life of mature adipocytes. Accordingly, PREF-1 decreased for controls and all treated cells, whereas PPARγ was expressed for all cells at day 4 (Figure 2 and Figure 3), confirming that adipogenesis was not affected by ellagic acid and urolithins A and B.

**Figure 4:**
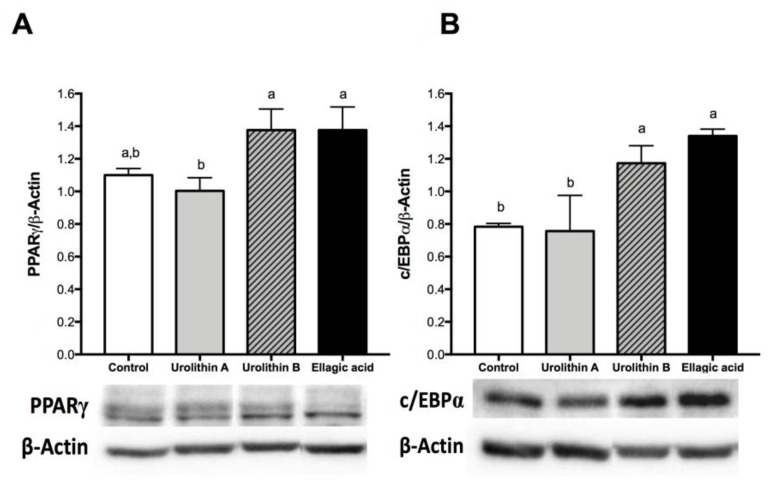
Effect of ellagic acid, urolithin A, and urolithin B in the protein expression of PPARγ (**A**) and CCAAT-enhancer-binding protein alpha (c/EBPα) (**B**) in 3T3-L1 adipocytes. The protein expression of PPARγ and c/EBPα were determined by Western blot after 8 days of treatment with 25 µM of the compounds, as described in the Materials and Methods section. The values represent the mean ± SD (*n* = 3) of three independent experiments. Different letters among bars denote significant changes among treatments and control (*p* ≤ 0.05) performed by ANOVA and Tukey’s post hoc test.

On the late stage, differentiation of cultured 3T3-L1 preadipocytes into adipocytes is accompanied by a dramatic rise in the rate of transcription of adipose-specific proteins, like PPARγ and c/EBPα [24]. On mature adipocytes (day 8), we found that 25 μM treatments with ellagic acid and urolithin B significantly up-regulated protein expression levels of PPARγ and c/EBPα. No significant changes compared with the control were observed for PPARγ and c/EBPα protein expression in cells treated with urolithin A (Figure 4). PPARγ has been proposed as the master regulator of adipogenesis, which is supported by evidence from both in vivo and in vitro studies. PPARγ can induce adipocyte differentiation in c/EBPα-deficient mouse embryonic fibroblasts, whereas c/EBPα is incapable of inducing the adipogenic program in the absence of PPARγ [18]. This observation suggests that c/EBPα and PPARγ participate in a single pathway of adipose development, in which PPARγ is the most important transcription factor. Several studies have shown that the activation of PPARγ in adipocytes can induce insulin-sensitization and restore the insulin-mediated glucose uptake, storage, and metabolism [25]. In contrast, Armoni et al. [26] reported that PPARγ represses glucose transporter type 4 (GLUT4). Moreover, PPARγ is able to activate c/EBPα, and c/EBPα is required for the induction of insulin sensitivity in adipocytes. This suggests that the insulin sensitization induced by PPARγ is in part mediated by c/EBPα activation. On the other hand, in addition to controlling insulin action, c/EBPα is required for maintaining expression of PPARγ in the mature fat cell [27], and both are considered key regulators in fatty acid biosynthesis and accumulation [28]. On the basis of the intracellular triglyceride accumulation results, we were expecting a decrease in c/EBPα and PPARγ protein expression after the treatments with ellagic acid and urolithin A. However, the present results did not show a clear relationship between these markers and the content of intracellular triglycerides. This fact suggests that these compounds could be modifying pathways related with PPARα and lipo-oxidation.

### 2.4. Effect of Ellagic Acid and Urolithins A and B on PPARα and Glycerol Release

**Figure 5:**
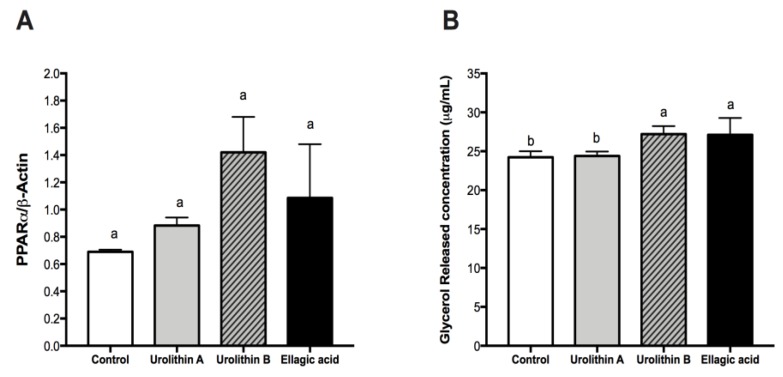
Effect of ellagic acid, urolithin A, and urolithin B in the protein expression of peroxisome proliferator-activated receptor-α (PPARα) (**A**) and measurement of glycerol released into the culture medium of 3T3-L1 adipocytes (**B**). The protein expression of PPARα was determine by Western blot after 8 days of treatment with 25 µM of the compounds, as described in the Materials and Methods section. The values represent the mean ± SD (*n* = 3) of three independent experiments. (B) Shows the concentration of glycerol released into the culture medium determined by spectrophotometry at 540 nm. Different letters among bars denote significant changes among treatments and control (*p* ≤ 0.05) performed by ANOVA and Tukey’s post hoc test.

After analyzing the effect on intracellular triglyceride content and adipocyte differentiation of ellagic acid and urolithins, we evaluated if these compounds modified the amount of glycerol released and protein expression of PPARα. In the adipose tissue, triglycerides stored in the intracellular lipid droplets can be hydrolyzed into free fatty acids (FA) and glycerol, which are subsequently released into the surrounding environment. Several enzymes responsible of this process are regulated by PPARα [29,30]. After treatments with ellagic acid and urolithins A and B in adipocytes from day 0 to day 8, we found that neither treatment affected significantly the protein expression level of PPARα (Figure 5A), however, the concentration of glycerol released into the culture medium was significantly increased with ellagic acid and urolithin B, but not urolithin A (Figure 5B).

PPARα is a dietary lipid sensor, whose activation results in hypolipidemic effects in vivo. In addition, its activation promotes both adipocyte differentiation and FA oxidation in 3T3-L1 adipocytes [29]. Our results did not show a direct relationship between PPARα activation and increased FA oxidation, suggesting that ellagic acid and urolithin B do not have PPARα agonist action, despite non-significant increased PPARα levels. PPARα activators are able to attenuate adiposity in animal models of obesity and type 2 diabetes mellitus [31,32], and PPARα agonists such as “fibrates” decrease circulating lipid levels and are commonly used to treat hyperlipidemia and other dyslipidemic states [31]. On the basis of the present findings, ellagic acid and urolithin B could be explored in the future as a natural alternative to fibrates, but further work is needed to confirm the mode of action of these compounds. In summary, in this part of the study, we found that urolithin A is a compound able to reduce intracellular triglyceride accumulation without affecting the protein expression of PPARγ, c/EBPα, and PPARα, as well as FA oxidation. On the other hand, we found that ellagic acid and urolithin B have a similar effect over the protein expression of PPARγ, c/EBPα, and PPARα, as well as FA oxidation, despite its differentiated effect over intracellular triglyceride accumulation. The present results suggest that these compounds probably act directly over enzymes related with FA oxidation or glucose uptake and metabolism. In the following section, studies on lipogenesis and lipolysis were conducted to understand the different mode of action for these compounds.

### 2.5. Effect of Ellagic Acid and Urolithins A and B on Adipokines, Lipogenesis, and Lipolysis

**Figure 6:**
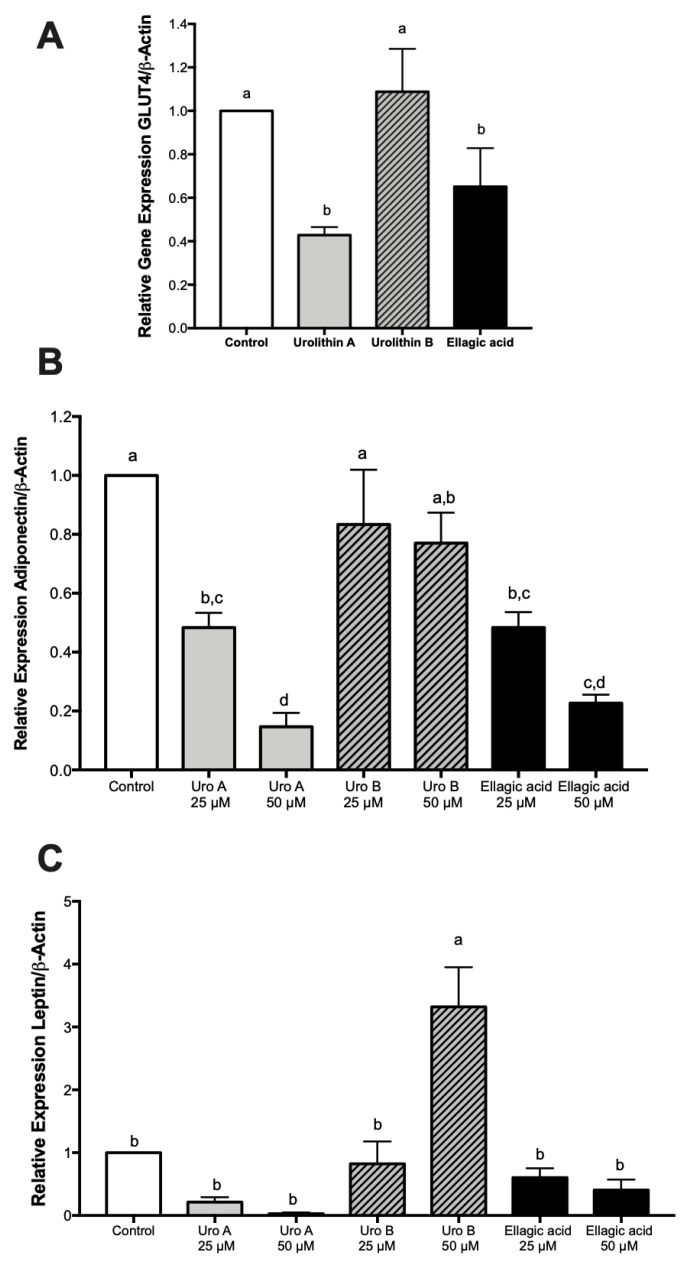
Effect of ellagic acid, urolithin A, and urolithin B on the gene expression of glucose transporter type 4 (GLUT4), adiponectin, and leptin in 3T3-L1 adipocytes. The gene expression of GLUT4 with treatments at 25 µM (**A**), and gene expressions of adiponectin (**B**) and leptin with treatments at 25 and 50 µM (**C**) was determined by RT-PCR after 8 days of treatment with the compounds, as described in the Materials and Methods section. The values represent the mean ± SD (*n* = 3) of three independent experiments. Different letters among bars denote significant changes among treatments and control (*p* ≤ 0.05) performed by ANOVA and Tukey’s post hoc test.

Fat accumulation is the result of the balance between lipogenesis and lipolysis events. Thus, we herein report the activities of different key players as affected by the treatments. Initially, we measured the gene expression of GLUT4 and regulatory adipokines adiponectin and leptin. Results showed a similar pattern among GLUT4 and adiponectin gene expressions with down-regulations for ellagic acid and urolithin A and no effects by urolithin B (Figure 6A,B) similar to the trend observed for fat accumulation (Figure 1C). On the other hand, neither of the compounds affected leptin gene expression with the exception of urolithin B at a higher dose (Figure 6C).

Furthermore, because PPARγ and c/EBPα regulate the genes involved in adipogenesis and lipogenesis, including adipocyte protein 2 (AP2), fatty acid synthase (FASN), stearoyl-CoA desaturase-1 (SCD1), and acetyl-CoA carboxylase-1 and 2 (ACC1 and ACC2), we measured their effects by ellagic acid and urolithins. Results indicated that urolithin A reduced mRNA levels of lipogenic genes AP2, ACC1, ACC2, FASN, and SCD1, whereas urolithin B down-regulated these genes with the exception of AP2 and SCD1. On the other hand, ellagic acid differentially affected these genes by down-regulating ACC1 and FASN, not affecting AP2, and up-regulating ACC2 and SCD1 (Figure S1).

Lipolysis regulates adipose tissue weight and obesity through enzymes that catabolize triglycerides, including hormone-sensitive lipase (HSL), adipose triglyceride lipase (ATGL), and lipid droplet-associated protein (perilipin). In mature adipocytes, urolithins A and B and ellagic acid decreased the transcriptional activities of these genes involved in lipolysis and the oxidative pathways, with the exception of ATGL for urolithin B and perilipin for ellagic acid (Figure S1).

Accordingly, these results suggest that lipid accumulation by ellagic acid and urolithins A and B is likely due to the regulation of the GLUT4 gene, which might be associated to the regulatory effects of adiponectin [33]. Thus, a decrease in GLUT4 leads to a decrease in glucose transport into the cells from the extracellular environment, reducing a key substrate for triglyceride biosynthesis [17]. In addition to the limitation of the key substrate necessary for fat biosynthesis, the differential effects of ellagic acid and urolithins A and B on gene expression of enzymes involved in lipogenesis and lipolysis, as observed above, will determine the rate kinetics of lipogenesis (k_Lg_) and lipolysis (k_Ls_), ultimately defining the fat accumulation in mature adipocytes. Thus, for ellagic acid and urolithin A, treatments that showed a reduction in fat accumulation the rate kinetics would be k_Ls_ > k_Lg_, whereas for the urolithin B treatment associated with fat accumulation, the rate kinetics would be k_Lg_ > k_Ls_. Further studies are needed to understand how ellagic acid and urolithn A regulate adiponectin levels and thus GLUT4, as well as the possible alternative mode of action to PPARγ that takes place.

### 2.6. Effect of Ellagic Acid and Urolithins A and B on LPS-Induced Inflammation and Insulin Sensitivity

Mature adipocytes challenged with LPS showed a significant increase in pro-inflammatory genes including tumor necrosis factor alpha (TNFα), interleukin 6 (IL-6), inducible nitric oxide synthase (iNOS), and monocyte chemoattractant protein-1 (MCP-1) while not affecting cyclooxygenase 2 (COX-2) (Figure S2). When LPS-challenged mature adipocytes were treated previously with urolithins A and B and ellagic acid, we observed a differential response, with down regulations of gene expressions for TNFα by urolithin A and ellagic acid, down regulations of iNOS for urolithins A and B and ellagic acid, and down regulation of IL-6 and MCP-1 only for ellagic acid. Treatments did not have major effects on COX-2.

**Figure 7:**
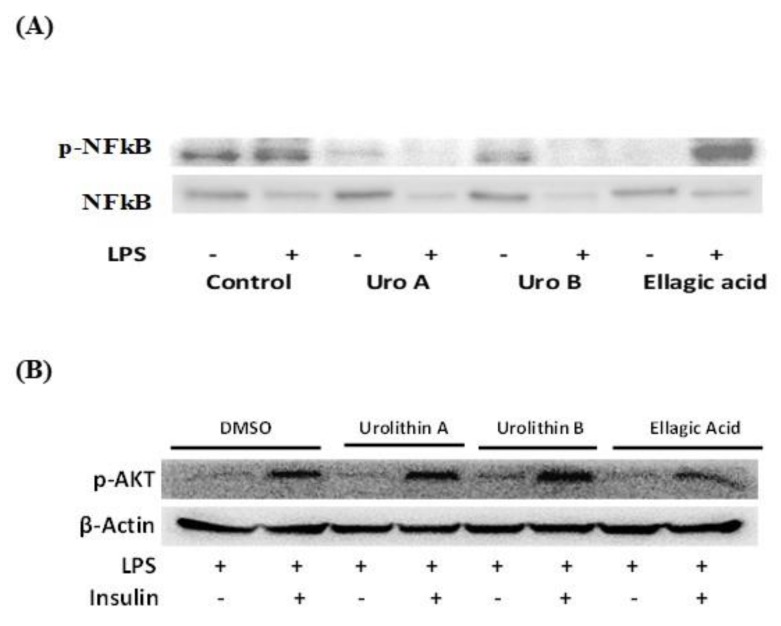
Ellagic acid and urolithins A and B effects on protein expression of transcription factors nuclear factor κB (NF-κB) and protein kinase B (AKT) associated with inflammation and insulin sensitivity in 3T3-L1 adipocytes at day 8 treated with compounds for 24 h and then exposed for 1 h to lipopolysaccharide (LPS; 100ng/mL). Western blot assays for nuclear phosphorylated nuclear factor κB (p-NF-κB; nuclear extract) and cytosolic NF-κB (cytosolic extract) at 25 µM treated adipocytes challenged with or without LPS (**A**) and nuclear phosphorylated AKT (p-AKT; nuclear extract) under LPS-induced inflammation in mature adipocytes with or without insulin (10 µg/mL) (**B**), as described in the Materials and Methods section.

To have an insight of the mode of action of the differential anti-inflammatory response observed, we evaluated the protein expression of transcription factor nuclear factor κB (NF-κB). Western blot assays showed a decrease in protein expression of phosphorylated nuclear factor κB (p-NF-κB) in nuclear extracts for urolithins A and B on adipocytes challenged with or without LPS, whereas for ellagic acid, this effect was only seen in non-LPS-challenged cells (Figure 7A). Accordingly, the anti-inflammatory properties observed for urolithins A and B and ellagic acid might be mediated by regulation of additional transcriptional factors besides p-NF-κB, for instance activator protein 1 (AP1); however, further studies are recommended to confirm this.

In addition, we measured the effects of ellagic acid and urolithins A and B on phosphorylated protein kinase B (p-AKT) under LPS-induced inflammation in adipocytes. Results indicated that urolithins A and B and ellagic acid-treated adipocytes showed expression of p-AKT in nuclear extracts under LPS challenge and stimulated by insulin (Figure 7B). This insulin response confirmed that ellagic acid and metabolites urolithins A and B do not affect insulin sensitivity in adipocytes.

Taken together, our results with murine cells indicate that EA and urolithin A may work potentially in different fronts by attenuating lipogenesis and fat accumulation without affecting adipogenesis, whereas only EA and urolithin B enhanced FA oxidation. On the other hand, EA and urolithins A and B differentially attenuated inflammation in mature adipocytes while not affecting insulin sensitivity. Other studies with primary human adipocytes later confirmed that urolithin A reduced fat accumulation but mainly through inhibition of adipogenesis and enhanced FA oxidation [34]. Nevertheless, considering the existant interindividual variability to produce urolithins and their different predispositions to chronic diseases, such as the metabolic syndrome [7], the present results support the idea that metabotype A individuals (urolithin A producers) may benefit from this dual role of urolithin A of reduced fat accumulation and decreased inflammation, compared to metabotype B individuals (mainly urolithin B producers) and metabotype 0 individuals (nonproducers), who are more susceptible to the metabolic syndrome. Age is considered the key factor in determining the gut microbiota involved in ellagic tannin–ellagic acid metabolism and ultimately the urolithin metabotypes, where younger individuals are type A and older individuals are mainly type B, with type 0 unaltered with age [35]. This opens the possibility of designing tailored diets and nutrition, as well as perhaps the microbiota of individuals. However, studies are still in need to better understand the specific roles of EA and derived urolithins in future works, or even to elucidate previous reports where EA might be the bioactive compound or alternatively the derived gut microbial urolithins. For instance, Yoshimura et al. [36] reported that EA improved hepatic steatosis and lipid composition through reduction of adipokine resistin and activation of PPARα in obese mice, and thus a next step would be to understand if EA or the derived urolithins are responsible for this effect.

## 3. Materials and Methods

### 3.1. Chemicals

The following chemicals were used in the experiments: 3-isobutyl-1-methylxanthine, dexamethasone, insulin, Dulbecco’s modified Eagle’s medium, fetal bovine serum (FBS), trypsin-EDTA, and protease inhibitor cocktail were purchased from Sigma (St. Louis, MO, USA). D-glucose was obtained from Acros Organics (Fair Lawn, NJ, USA). Sodium bicarbonate was purchased from Mallinckrodt Chemicals (Phillipsburg, NJ, USA). Murine 3T3-L1 preadipocytes and dimethyl sulfoxide (DMSO) were acquired from the American Type Culture Collection (ATCC) (Manassas, VA, USA). Penicillin-streptomycin was bought from Invitrogen (Carslbad, CA, USA). Urolithins A and B were manufactured by Kylolab (Ceuti, Spain). Ellagic acid was from MP Biomedicals (Solon, OH, USA). Cell lysis buffer was obtained from Cell Signaling Technology (Danvers, MA, USA). Sodium dodecyl sulfate solution, 30% acrylamide/bisacrylamide solution, *N*,*N*,*N*′,*N*′-tetramethylethylenediamine (TEMED), ammonium persulfate, Tween 20, and Precision Plus Protein marker were obtained from Bio-Rad Laboratories (Hercules, CA, USA). Laemmli’s loading buffer was acquired from Fermentas Inc. (Glen Burnie, MD, USA). Polyvinylidene fluoride (PVDF) membranes were obtained from Millipore Corp. (Billerica, MA, USA). Antibodies for PPARγ (sc-7196), c/EBPα (sc-61), PPARα (sc-9000), p-NF-κB (sc-33039), p-AKT, and β-actin (sc-47778) were purchased from Santa Cruz Biotechnology (Santa Cruz, CA, USA). Antibody for NF-κB (C22B4) was obtained from Cell Signaling Technology (Danvers, MA, USA). Goat anti-rabbit-horseradish peroxidase (HRP) polyclonal secondary antibody (A120-101P) was obtained from Bethyl Laboratories (Montgomery, TX, USA).

### 3.2. Cell Culture

Murine 3T3-L1 preadipocytes (ATCC, Manassas, VA, USA) were maintained in high glucose Dulbecco’s modified Eagle’s medium (DMEM) supplemented with 10% fetal bovine serum (Sigma, St. Louis, MO, USA), penicillin (100 unit/mL), and streptomycin (100 μg/mL), and incubated under humidified atmosphere at 37 °C and 5% CO_2_.

### 3.3. Cell Viability Assay

Cell viability on murine 3T3-L1 preadipocytes was determined using MTS assay (Promega Corp., Madison, WI, USA), according to the manufacturer’s instructions. The cells were seeded at a density of 7500 cells per well in a 96-well plate and incubated with 10% FBS/DMEM medium for 24 h. Treatments with ellagic acid, urolithin A, and urolithin B were added at a concentration of 25 µM in DMSO. Final DMSO concentration in the culture medium was 0.025%; DMSO was added at this percentage as control. Cell viability was measured at 24 h; absorbance was measured at 490 nm in a microplate reader (Synergy HT, Bio-Tek Instruments, Inc., Winooski, VT, USA).

### 3.4. Cell Differentiation and Treatments for Adipogenesis Assays

Murine 3T3-L1 cells were seeded at a density of 10,000 cells per well in 6-well plates. Preadipocytes were induced to differentiation 2 days after they reached 100% confluency (day 0). Growth medium was supplemented with 0.5 mM 3-isobutyl-1-methylxanthine (IBMX), 1 μM dexamethasone, and 10 μg/mL insulin, for 48 h. Additionally, on day 0, treatments of ellagic acid, urolithin A, and urolithin B were added to reach a final concentration of 25 µM, and diluted in DMSO. At day 2, medium was replaced with fresh medium supplemented with insulin (10 μg/mL) for 2 additional days, until day 4. After day 4, 10% FBS/DMEM medium was replaced every 2 days until >90% fully mature adipocytes were reached (day 8).

### 3.5. Quantification of Lipid Content

Intracellular lipid content was measured using a commercially available AdipoRed kit (AdipoRed, Lonza Wakersville, Inc., Wakersville, MD, USA). AdipoRed is a solution of the Nile Red stain, which fluoresces and enables the quantification of intracellular lipid droplets. Preadipocytes were differentiated and treated following the method previously described. Briefly, on day 8, cells were washed with 2 mL of phosphate-buffered saline (PBS, pH 7.4); then, 5 mL PBS was left per well and 140 μL AdipoRed was added, the plate was left at 37 °C for 10 min, and fluorescence readings were measured by well scanning with excitation at 485 nm and emission at 560 nm in a plate reader (Synergy HT, Bio-Tek Instruments, Inc., Winooski, VT, USA). Measurements were expressed as relative fluorescence units (RFU).

### 3.6. Quantification of Glycerol Release

The amount of glycerol released into the culture medium was determined using the Glycerol Cell-Based Assay Kit of Cayman Chemical Company (item 10011725, Ann Arbor, MI, USA), following the manufacturer’s instructions. In this assay, the amount of glycerol released into the medium is proportional to the triglyceride/fatty acid cycling rate. To prepare the cells for this assay, we seeded 3T3-L1 preadipocytes at a density of 8500 cells per well in 12-well plates. Two days after cells reached 100% confluency (day 0), we induced differentiation, following the method described previously. Ellagic acid, urolithin A, and urolithin B were co-incubated with the cells at a concentration of 25 µM from day 0 to day 8. At day 8, the cells were washed and the glycerol cell-based assay was performed. The concentration of glycerol released was determined by absorbance at 540 nm in a plate reader (Synergy HT, Bio-Tek Instruments, Inc., Winooski, VT, USA).

### 3.7. Ellagic Acid and Urolithins A and B on LPS-Induced Inflammation and Insulin Sensitivity

The effect of ellagic acid and urolithins A and B on the activation of p-NF-κB and the gene expression of several inflammation markers including TNFα, IL-6, COX-2, iNOS, and MCP-1 was studied in mature adipocytes (day 8). Fully mature adipocytes (obtained as described above) were exposed for 24 h to 25 and 50 μM of ellagic acid, and urolithins A and B, and then treated with LPS (100 ng/mL) for 1 h to induce the inflammatory response. To test insulin sensitivity, mature adipocytes were obtained as described above and treated with ellagic acid and urolithins A and B with 25 μM for 24 h, where they were then exposed to LPS (100 ng/mL) and insulin (10 μg/mL) for 1 h. Protein and mRNA samples were collected and stored at −80 °C until used.

### 3.8. Western Blot Analysis

Cells were lysed on ice using cell lysis buffer (Cell Signaling Technology, Danvers, MA, USA) supplemented with protease and phosphatase inhibitors, following the manufacturer’s instructions. Briefly, cells were then scraped, left at −80 °C overnight, centrifuged at 14,000 rpm at 4 °C, and the supernatant was stored at −80 °C. The protein concentration was determined using the BCA Protein Assay Kit (Pierce, Thermo Fisher Scientific, Inc., Rockford, IL, USA). Cell protein from nuclear and cytosolic extract was obtained with a nuclear extraction kit (Cayman Chemical, item 10009277, Ann Arbor, MI, USA) following the manufacturer’s instructions. In our experiments, 40 µg of protein was loaded. Equal amounts of proteins were separated by SDS-polyacrylamide gels and then electrophoretically transferred from the gel onto a PVDF membrane (Millipore, Bedford, MA, USA). The membranes were then blocked with 5% non-fat milk in Tris-buffered saline with 1% Tween-20 (TBS-T) for 1 h with gentle shaking; washing four times (5 min) with TBS-T was performed consecutively. Membranes were then incubated with a specific primary antibody against PPARγ (1:1000), PPARα (1:5000), c/EBPα (1:7000), p-NF-κB (1:2000), NF-κB (1:1500), and p-AKT (1:1000). For internal control, membranes were incubated with a conjugated HRP primary antibody against β-actin at a dilution of 1:10,000. The membranes were washed four times (5 min) with TBS-T and incubated for 1 h with the secondary antibody conjugated with horseradish peroxidase (HRP) at 1:30,000 dilutions. The blots were developed using a SuperSignal West Femto enhanced chemiluminescence (ECL) Western blotting detection kit (Pierce, Thermo Fisher Scientific, Inc., Rockford, IL, USA) after signals were captured by CCD Camera (Cascade II:512, Photometric, Tucson, AZ, USA) using the Win View/32 software (Version 2.5, Princeton Instruments, Trento, NJ, USA). The blot images were quantitated by densitometry using the Image J software (NIH, Bethesda, MD, USA).

### 3.9. Real-Time Quantitative RT-PCR

Total RNA was extracted from 3T3-L1 mature adipocyte controls and treatment at 25 and 50 µM with ellagic acid and urolithins A and B using the RNeasy mini kit (Qiagen, Valencia, CA, USA) in accordance with the manufacturer’s instructions. RNA concentration was measured with a NanoDrop ND-1000 spectrophotometer (NanoDrop Technologies, Willmington, DE, USA). One microgram of RNA was reverse-transcribed into cDNA using the SuperScript III first-strand synthesis supermix (item 18080-004, Invitrogen, Carlsbad, CA, USA), following the manufacturers protocol. The real-time PCR for leptin was quantified using Power SYBR Green PCR Master Mix (Applied Biosystems, Foster City, CA, USA), following the manufacturer’s instructions. DNA amplification was carried out using a 7900 HT Sequence Detection System (Applied Biosystems, Foster City, CA, USA). Real time PCR reaction was performed in a 20 μL volume containing Power SYBR Green PCR Master Mix and 25 ng cDNA, and 0.3 µM primers mouse leptin 5’-GAGACCCCTGTGTCGGTTC-3’ (forward) and 5’-CTGCGTGTGTGAAATGTCATTG-3’ (reverse); mouse β-actin 5’-CCCAGGCATTGCTGACAGG-3’ (forward) and 5’-TGGAAGGTGGACAGTGAGGC-3’ (reverse); mouse ACC1 5′-GGATGGTTTGGCCTTTCACA-3′ (forward) and 5′-TTTTCTTTCTGTCTCGACCTTGTTT-3′ (reverse); mouse ACC2 5′-ACAGAGATTTCACCGTTGCGT-3′ (forward) and 5′-CGCAGCGATGCCATTGT-3′ (reverse); mouse AP2 5′-CTTCAAACTGGGCGTGAA-3′ (forward) and 5′-CTAGGGTTATGATGCTCTTCACCTT-3′ (reverse); mouse FASN 5′-GGCTCAGCATGGTCGCTT-3′ (forward) and 5′-CTCCCGCCAGCTGTCATT-3′ (reverse); mouse GLUT4 5′-TTGGTACCTACGCTTTGCAGC-3′ (forward) and 5′-CGGTTAGAGCGCATCAGTCTC-3′ (reverse); mouse HSL 5′-GCAAGATCAAAGCCTCAGCG-3′ (forward) and 5′-GCCATATTGTCTTCTGCGAGTGT-3′ (reverse); mouse ATGL 5′-GTCCTTCACCATCCGCTTGTT-3′ (forward) and 5′-CTCTTGGCCCTCATCACCAG-3′ (reverse); mouse SCD1 5′-ATCGCCTCTGGAGCCACAC-3′ (forward) and 5′-ACACGTCATTCTGGAACGCC-3′ (reverse); mouse perilipin 5′-GGTACACTATGTGCCGCTTCC-3′ (forward) and 5′-CTTTGCGCTCCGCCTCT-3′ (reverse); mouse PREF-1 5′-CAG CGGCTATGGGCTCACCT-3′ (forward) and 5′-TGTTGCTCGGGCTGCTGAA-3′ (reverse); mouse TNFα 5’-ACTGGCAGAAGAGGCACTCC-3’ (forward) and 5’-CGATCACCCCGAAGTTCA-3’ (reverse); mouse IL-6 5’-TGACAACCACGG CCTTCCCT-3’ (forward) and 5’-AGCCTCCGACTTGTGAAGTGGT-3’ (reverse); mouse COX-2 5’-ACATCGATGTCATGGAACTG-3’ (forward) and 5’-GGACACCCCTTCACATTATT-3’ (reverse); mouse iNOS 5’-ACATCGACCCGTCCACAGTAT-3’ (forward) and 5’-CAGAGGGGTAGGCTTGTCTC-3’ (reverse); mouse MCP-1 5’-CAGCCAGATGCAATCAATGC-3’ (forward) and 5’-GTGGTCCATGGAATCCTGAA-3’ (reverse) were provided by Integrated DNA Technologies (IDT, Coralville, IA). After 10 min polymerase activation at 95 °C, 40 cycles with 95°C for 15 s (denaturation) and 60 °C for 1 min (annealing/extension) were performed. Fluorescence was measured at the end of the 60 °C extension period. The relative expression of genes was normalized using β-actin, and was calculated following the comparative Ct method (ΔΔCt), also known as the 2^−ΔΔCt^ method [37].

### 3.10. Statistical Analysis

Data represent the mean ± SD for cell viability assay and all the other markers measured related with adipogenesis, lipogenesis, lipolysis, and inflammation. Statistical significance was assessed by ANOVA and separation of means by Tukey’s post hoc test. Differences were considered significant when *p* ≤ 0.05. Tests were conducted using JMP, Version 15 (SAS Institute Inc., Cary, NC, USA, 1989–2019).

## 4. Conclusions

Ellagic acid and urolithins A and B did not affect adipogenesis, however, they differentially affected lipid accumulation. Although urolithin A and ellagic acid decreased fat accumulation in adipocytes, urolithin B showed similar lipid accumulation to that of controls. This differential response seemed to be associated with the regulation of GLUT4 gene expression and associated changes in adiponectin that followed similar trends.

In addition, we report that ellagic acid and urolithins A and B showed anti-inflammatory properties in LPS-challenged mature adipocytes, and this response was differentially observed in gene expressions of pro-inflammatory markers including TNF-α, IL-6, iNOS, and MCP-1. This response was in part associated with a decrease in nuclear transcription factor p-NF-κB and possibly to the regulation of other transcription factors, for instance, AP1. Furthermore, we report that nuclear transcription factor p-AKT was expressed in mature adipocytes treated with ellagic acid and urolithins A and B when challenged with LPS and exposed to insulin, confirming the idea that mature adipocytes are sensitive to insulin.

**Figure 8:**
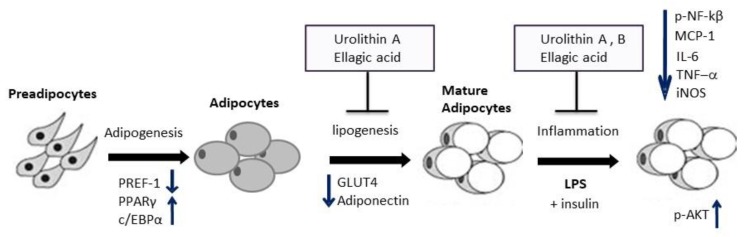
Proposed model of modulation of lipogenesis and inflammation by ellagic acid and derived gut microbial metabolites urolithins A and B in adipocytes. Ellagic acid (EA) and urolithins A and B did not affect adipogenesis in 3T3-L1 cells, where PREF-1 decreased and PPARγ and c/EBPα increased for all treatments. During lipogenesis/lipolysis, only EA and urolithin A reduced fat accumulation in mature adipocytes attenuating GLUT4 gene expression and adiponectin. In LPS-challenged cells, EA and urolithins A and B differentially ameliorated inflammation through down-regulation of transcription factor p-NF-κB and pro-inflammatory genes while not affecting insulin sensitivity, depicted by an increase in protein expression of transcription factor p-AKT when exposed to insulin.

In general, our study gives insight into the mode of action of how ellagic acid and derived gut microbial metabolites urolithins A and B differentially attenuate lipid accumulation and inflammation in mature adipocytes (Figure 8). This information is relevant for crops such as pecans and others that contain high levels of ellagitannins [38]. Further efforts are recommended for in in vivo studies to confirm these results.

## Supplementary Materials

Supplementary materials can be found at https://www.mdpi.com/1422-0067/21/6/2086/s1.

## Abbreviations

ACC1	acetyl-CoA carboxylase 1
ACC2	acetyl-CoA carboxylase 2
AKT	protein kinase B
AP1	activator protein 1
AP2	adipocyte protein 2
ATGL	adipose triglyceride lipase
c/EBPα	CCAAT-enhancer-binding protein alpha
COX-2	cyclooxygenase 2
DMSO	dimethyl sulfoxide
EA	ellagic acid
FASN	fatty acid synthase
GLUT4	glucose transporter type 4
HSL	hormone sensitive lipase
IL-6	interleukin 6
iNOS	inducible nitric oxide synthase
LPS	lipopolysaccharide
MCP-1	monocyte chemoattractant protein-1
NF-κB	nuclear factor κB
p-AKT	phosphorylated protein kinase B
p-NF-κB	phosphorylated nuclear factor κB
PPARα	peroxisome proliferator-activated receptor alpha
PPARγ	peroxisome proliferator-activated receptor gamma
SCD1	stearoyl-CoA desaturase
TNFα	tumor necrosis factor alpha
Uro	urolithin
