# Supplementary material for: Ellagic Acid and Urolithins A and B Differentially Regulate Fat Accumulation and Inflammation in 3T3-L1 Adipocytes While Not Affecting Adipogenesis and Insulin Sensitivity
Source: Int J Mol Sci. 2020 Mar 18;21(6):2086. doi: 10.3390/ijms21062086 (PMC7139477; doi:10.3390/ijms21062086)
Supplement: Supplementary file 1 [file ijms-21-02086-s001.pdf]

International Journal of
Molecular Sciences

Supplementary Materials:

Ellagic Acid and Urolithins A and B Differentially Regulate Fat Accumulation and Inflammation in 3T3-L1 Adipocytes While Not Affecting Adipogenesis and Insulin Sensitivity

Luis Cisneros-Zevallos [1,2,*], Woo Young Bang [1] and Claudia Delgadillo-Puga [3]

[1] Department of Horticultural Sciences, Texas A&M University, College Station, TX 77843-2133, USA; wybang@gmail.com
[2] Department of Nutrition and Food Science, Texas A&M University, College Station, TX 77843, USA
[3] Departamento de Nutrición Animal Dr. Fernando Pérez-Gil Romo, Instituto Nacional de Ciencias Médicas y Nutrición Salvador Zubirán (INCMNSZ), CDMX 14080, Mexico; claudia.delgadillop@incmnsz.mx
* Correspondence: lcisnero@tamu.edu; Tel.: +1-979-845-3244

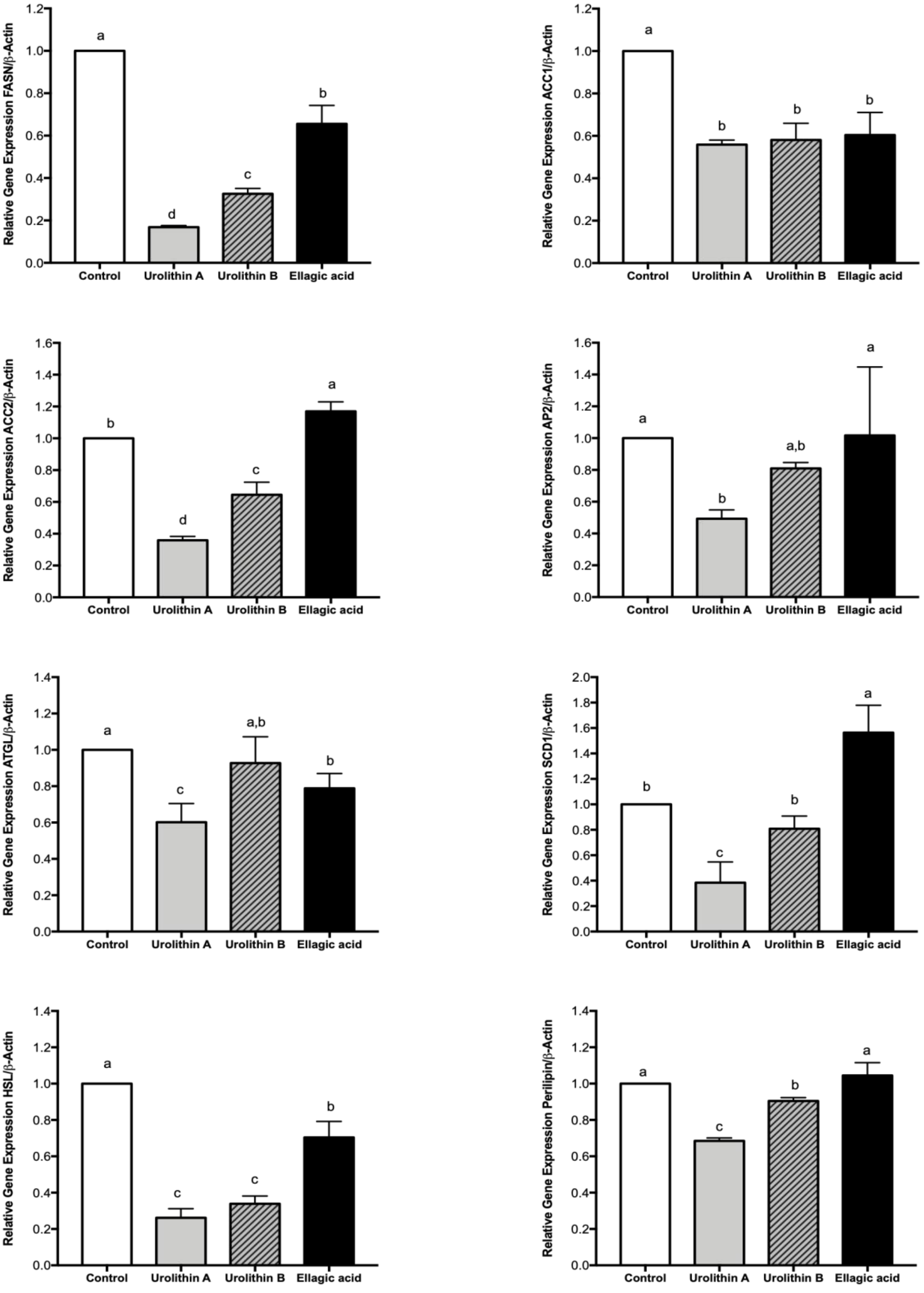

Figure S1. Gene expression of lipogenic (FASN, ACC1, ACC2, AP2, and SCD1) and lipolytic (HSL, ATGL and Perilipin) enzymes in mature 3T3-L1 adipocytes after 8-day treatment with 25 μM of urolithins A and B and ellagic acid. Gene expression was evaluated by RT-PCR as described in the Materials and Methods section. The values represent the mean ± S.D. (n = 3) of three independent experiments. Different letters among bars denote significant changes among treatments and control ($p \leq 0.05$) performed by ANOVA and Tukey's post hoc test.

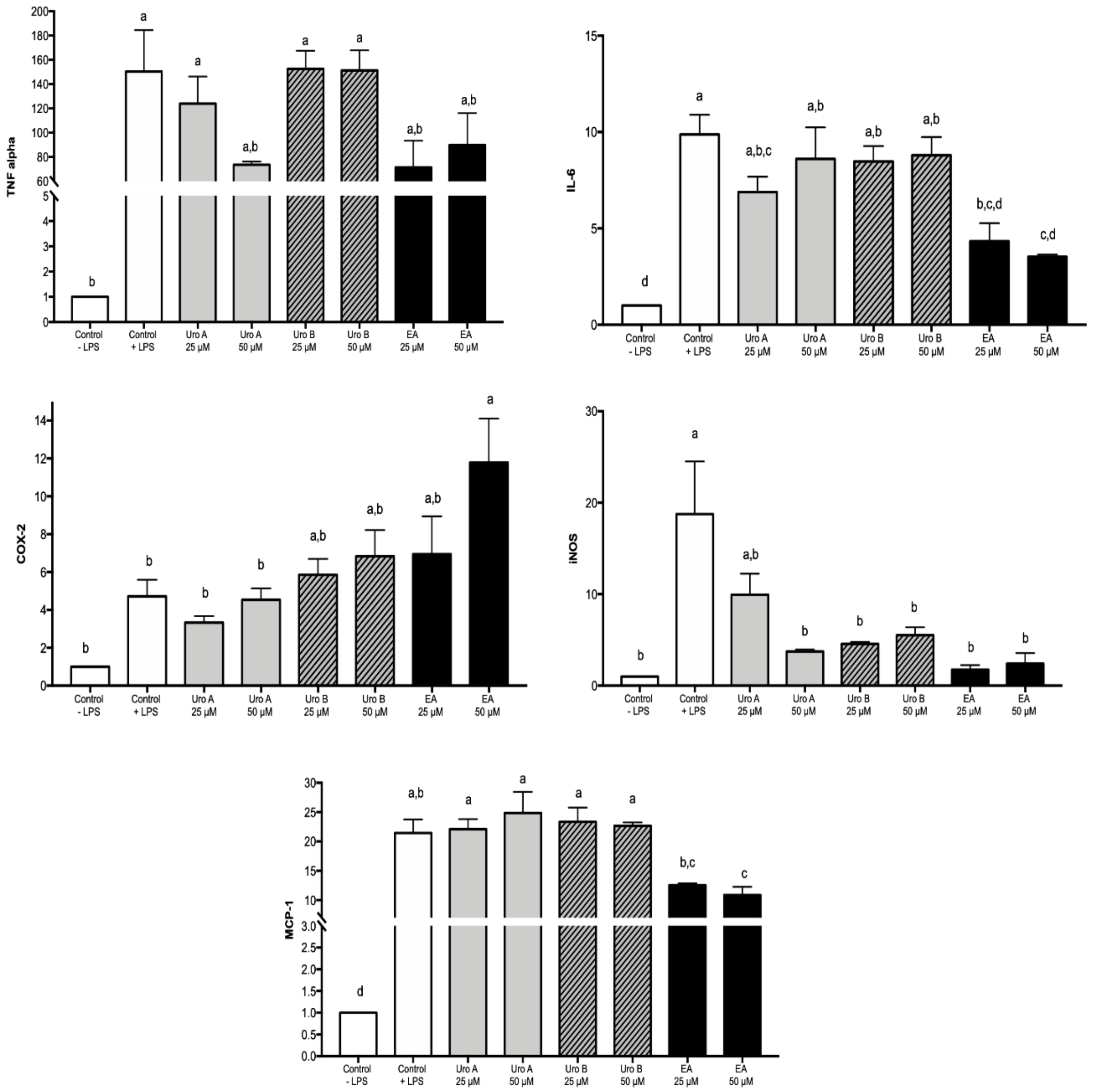

Figure S2. Gene expression of pro-inflammatory genes (TNF-α, IL-6, COX-2, iNOS and MCP-1) in LPS challenged mature 3T3-L1 adipocytes. Cells were treated with urolithins A and B and ellagic acid at concentrations of 25 µM and 50 µM, at day 8 for 24h and then exposed 1h to LPS (100 ng/mL). Gene expression was determined by RT-PCR as described in the Materials and Methods section. The values represent the mean ± S.D. (n = 3) of three independent experiments conducted in duplicate. Different letters among bars denote significant changes among treatments and control ($p \leq 0.05$) performed by ANOVA and Tukey's post hoc test.
